# The *Dictyostelium discoideum* homologue of Twinkle, Twm1, is a mitochondrial DNA helicase, an active primase and promotes mitochondrial DNA replication

**DOI:** 10.1186/s12867-018-0114-7

**Published:** 2018-12-19

**Authors:** Ashley Harman, Christian Barth

**Affiliations:** 1 Department of Physiology, Anatomy and Microbiology, La Trobe University, Melbourne, VIC Australia; 2 Present Address: Cell Biology Unit, Children’s Medical Research Institute, University of Sydney, Westmead, NSW Australia

**Keywords:** Twinkle, DNA helicase, DNA primase, Mitochondrial DNA replication, *Dictyostelium discoideum*

## Abstract

**Background:**

DNA replication requires contributions from various proteins, such as DNA helicases; in mitochondria Twinkle is important for maintaining and replicating mitochondrial DNA. Twinkle helicases are predicted to also possess primase activity, as has been shown in plants; however this activity appears to have been lost in metazoans. Given this, the study of Twinkle in other organisms is required to better understand the evolution of this family and the roles it performs within mitochondria.

**Results:**

Here we describe the characterization of a Twinkle homologue, Twm1, in the amoeba *Dictyostelium discoideum*, a model organism for mitochondrial genetics and disease. We show that Twm1 is important for mitochondrial function as it maintains mitochondrial DNA copy number in vivo. Twm1 is a helicase which unwinds DNA resembling open forks, although it can act upon substrates with a single 3′ overhang, albeit less efficiently. Furthermore, unlike human Twinkle, Twm1 has primase activity in vitro. Finally, using a novel in bacterio approach, we demonstrated that Twm1 promotes DNA replication.

**Conclusions:**

We conclude that Twm1 is a replicative mitochondrial DNA helicase which is capable of priming DNA for replication. Our results also suggest that non-metazoan Twinkle could function in the initiation of mitochondrial DNA replication. While further work is required, this study has illuminated several alternative processes of mitochondrial DNA maintenance which might also be performed by the Twinkle family of helicases.

**Electronic supplementary material:**

The online version of this article (10.1186/s12867-018-0114-7) contains supplementary material, which is available to authorized users.

## Background

DNA helicases are an important class of enzymes involved in many cellular processes. Their ability to unwind double-stranded DNA (dsDNA) allows the resulting single-stranded DNA (ssDNA) to serve as a template for downstream activity (e.g. DNA replication or repair) by other proteins or potentially the helicases themselves. During DNA replication, strand separation allows for the synthesis of a short RNA primer, which in turn is utilized by the DNA polymerase. While the processes of mitochondrial DNA (mtDNA) replication are similar to their nuclear counterparts, the proteins performing these roles often differ. The replicative DNA helicase in mitochondria is the highly conserved Twinkle family, which was originally identified by its homology to the bacteriophage T7 gp4 primase/helicase [1]. Twinkle is important for mitochondrial function, as mutations in its encoding gene in humans can cause autosomal dominant progressive external ophthalmoplegia, a neuromuscular disorder often associated with mtDNA deletions [1, 2].

Twinkle helicases function as 5′→3′ helicases and, similar to other replicative helicases, form ring-shaped hexamers to unwind dsDNA at the replication fork [3, 4]. Due to this structure, replicative helicases often require a loader protein to allow for the unwinding of DNA with no free 5′ end, which is typically the case for mtDNA [5, 6]. However, both T7 gp4 and human Twinkle are able to load themselves onto circular DNA without the need for accessory proteins [7]. The exact mechanism of this is unknown, although it has been theorized that the N-terminal domain of the protein is involved. While deletion of the N-terminal region in human Twinkle decreases mtDNA replisome activity in vitro, it is unclear whether this is solely due to its role in DNA binding and unwinding [3], or if the deletion also impairs loading of Twinkle onto a circular template. Furthermore, deletion of the T7 gp4 linker region (located between the primase and helicase domains) results in inefficient loading of the ring-shaped hexamer on DNA [8], which could also apply to Twinkle. This linker region is also important for the proper function and hexamerization of Twinkle [9].

Most Twinkle homologues are predicted to possess a primase domain N-terminally of their helicase domain, similar to the T7 gp4 primase/helicase [10]. While both *Arabidopsis thaliana* and *Plasmodium falciparum* encode homologues with functional primase domains [11–14], this domain is purportedly inactive in human Twinkle [3] and is theorized to be inactive in all metazoan Twinkle homologues [10]. Primase activity in Twinkle is also less well understood (than its helicase activity), however should this domain be active it is likely to prime ssDNA for the initiation of mtDNA replication [10]. While the primase inactivity of metazoan Twinkle could disprove this theory, the ability of the mitochondrial RNA polymerase to prime mtDNA has likely replaced or facilitated the loss of this activity in metazoan Twinkle [15, 16].

Research has largely focused on human Twinkle, meaning that little is known about the protein in non-metazoan eukaryotes. In various organisms, putative homologues have been identified bioinformatically; *A. thaliana* for example is predicted to encode at least three Twinkle homologues [10], one of which has been characterized in detail [11]. Interestingly, yeasts and fungi appear to have lost their Twinkle homologues [10], but also encode helicases only found in these lineages [17, 18]. Beyond multicellular eukaryotes, only PfPREX in *P. falciparum* has been identified as a replicative primase/helicase which targets to the apicoplast [12] and possesses an active primase domain [19]. While PfPREX is homologous to Twinkle, its open reading frame also encodes an active DNA polymerase domain [20], which may form a single polypeptide with the helicase. Given this protein’s putative structure, in conjunction with its apicoplast targeting, there is currently nothing known about mitochondrial Twinkle in unicellular organisms. Further studies outside humans would therefore better our understanding of the collective roles of the Twinkle protein family.

The social amoeba *Dictyostelium discoideum* is a well-established model organism for studying mitochondrial genetics and disease [21]. While previous research has investigated *D. discoideum* mitochondrial transcription [22, 23], little is known about the processes that govern its mtDNA maintenance. Here we describe the characterization of a Twinkle homologue (Twm1) in *D. discoideum*, encoded by the nuclear *twm1* gene and targeted to mitochondria. Twm1 is important in mitochondria, as antisense inhibition of its encoding gene leads to mitochondrial dysfunction and reduced mtDNA copy number. Heterologously expressed Twm1 possesses nucleoside triphosphatase (NTPase), helicase and, unlike human Twinkle, primase activity in vitro. Finally, using a novel in bacterio system, we demonstrated that Twm1 is capable of promoting DNA replication. Based on these findings we have concluded that Twm1 is a likely replicative mtDNA helicase in *D. discoideum* and a potential contributor to the initiation of mtDNA replication.

## Results

### *D. discoideum* Twm1 localizes to mitochondria

**Fig. 1:**
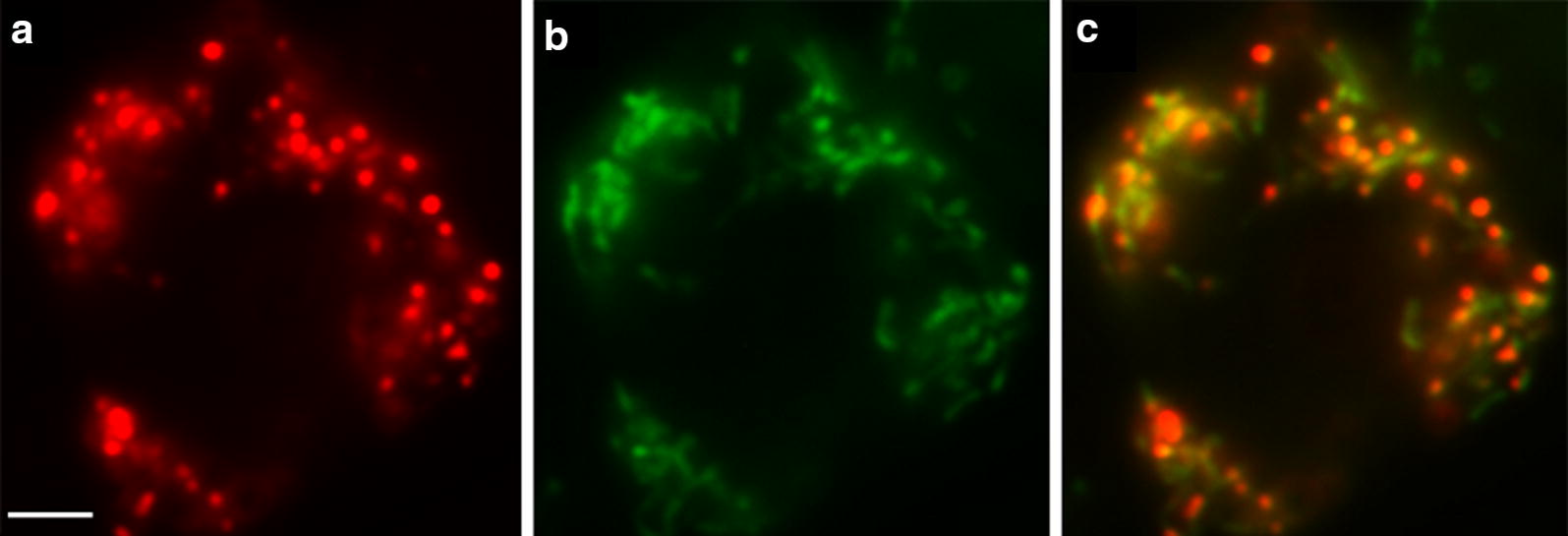
Mitochondrial localization of *D. discoideum* Twm1. Fluorescence microscopy of *D. discoideum* cells **a** stained with Mitotracker Red and **b** expressing a Twm1-GFP fusion protein **c** overlayed. Image is representative of the transformant population observed under ×1000 magnification with immersion oil. Scale bar = 5 µm

A gene encoding a putative Twinkle homologue in *D. discoideum* was previously identified by Shutt and Gray [10]. This gene, which we subsequently named *twm1*, contains no introns and encodes a 772 amino acid protein (accession no. XP_636842). In silico analysis using InterProScan [24] predicted this protein to contain a primase and a helicase domain (Additional file 1: Figure S1), both of which are conserved amongst Twinkle homologues and the T7 gp4 primase/helicase [10]. Given the homology of the *D. discoideum* protein to other Twinkle proteins, the subcellular localization of Twm1 was first examined. The predictive software packages Mitoprot and TargetP [25, 26] suggested the protein to be mitochondrially targeted (probability scores 0.9887 and 0.852, respectively). The subcellular localization of Twm1 was confirmed by creating a fusion gene within the *D. discoideum* expression vector pDV-CGFP [27]. The 5′ region of *twm1*, encoding the putative mitochondrial targeting signal, was cloned upstream of a GFP encoding gene and the resulting construct was transformed into *D. discoideum* cells. When visualized using fluorescence microscopy, the encoded Twm1-GFP fusion protein co-localized with stained mitochondria (Fig. 1), demonstrating that the targeting signal at the N-terminus of Twm1 directs the protein to mitochondria. From this we concluded that Twm1 is a mitochondrial protein.

### Antisense inhibition of *twm1* induces mitochondrial dysfunction and a reduction in mtDNA copy number

**Fig. 2:**
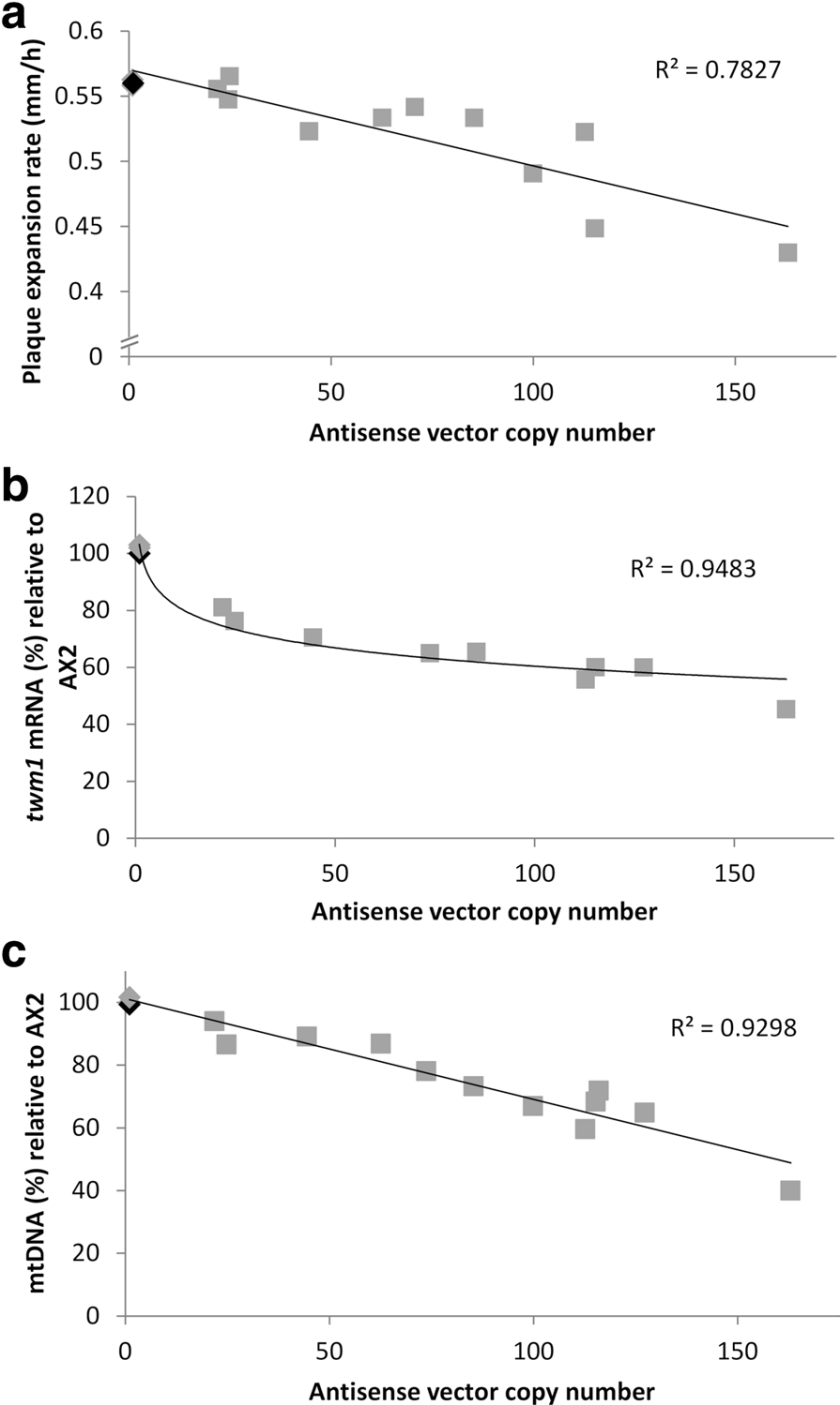
Effects of *twm1* antisense inhibition on *D. discoideum* mitochondrial function. **a** Plaque expansion rates of *twm1* antisense transformants on *E. coli* B2 lawns. Rates are plotted against antisense vector copy number, which was determined through qPCR. **b** Relative *twm1* mRNA levels in antisense transformants, quantified using qRT-PCR, normalized against a structural gene (*tubB*) and calculated as a percentage of the parental AX2. Values were plotted against antisense vector copy number. The *twm1* specific primers (Additional file 3: Table S1B) used recognize downstream of the region targeted by antisense inhibition to quantify only full length *twm1* mRNA. **c** mtDNA copy number of *twm1* antisense transformants. mtDNA copy number was determined through qPCR by comparing against a single copy nuclear gene, *tubB*. Antisense transformants were also compared to the parental AX2, and values plotted against antisense vector copy number. Parental AX2 is depicted in black, with transformants in grey; antisense transformants are shown as squares, while vector controls are diamonds. Vector controls were plotted ignoring vector copy number (102, 142 and 193) given there was no antisense inhibition

Given that members of the Twinkle protein family are thought to serve as replicative mtDNA helicases, it was suspected that *D. discoideum* Twm1 is equally important for mtDNA maintenance and overall mitochondrial function. The putative role of Twm1 was initially examined via antisense inhibition. In *D. discoideum*, transformation with vector DNA results in random integration at a single site within the genome and incorporation of a variable number of vector copies due to co-insertional replication [28]. As a consequence of this mechanism, each transformant possesses a different level of antisense inhibition. Following transformation of *D. discoideum* AX2 cells with the *twm1* antisense construct, transformants were isolated and their growth on bacterial lawns measured as an indicator of overall mitochondrial dysfunction. In *D. discoideum* mitochondrial dysfunction is known to trigger an inhibition of ATP consuming processes, such as growth on bacterial lawns [29, 30]. All of the *twm1* antisense transformants displayed slower plaque expansion rates than the parental strain (Fig. 2a). This growth defect also correlated well (R^2^ = 0.7827) with the vector copy number in each transformant, which was quantified by qPCR. The *twm1* mRNA level of each antisense transformant was also measured to confirm that transformation of the antisense construct produced altered gene expression in vivo. Compared to the parental AX2, *twm1* expression was reduced in all transformants, which also correlated with the vector copy number (Fig. 2b). Vector controls (copy numbers > 100) did not exhibit defective growth (Fig. 2a) or altered *twm1* mRNA levels (Fig. 2b), demonstrating that the observed growth defects are attributable to antisense inhibition of *twm1*, confirming a role for Twm1 in *D. discoideum* mitochondrial function.

Past work in metazoans has shown that overexpression or knockdown of Twinkle increases or decreases mtDNA copy number, respectively [31]. Therefore, the impact of *twm1* antisense inhibition on *D. discoideum* mtDNA was subsequently analyzed. Knockdown of any mtDNA maintenance machinery is likely to produce a decrease in mtDNA copy number [18], which is expected to be most pronounced when the expression of replicative protein genes is reduced [31]. To investigate this, *D. discoideum* AX2 and antisense transformants were grown to equal densities and total DNA was extracted. qPCR was used to compare the mtDNA in each transformant relative to both its nuclear DNA and AX2 mtDNA. All antisense transformants possessed less mtDNA than AX2 (Fig. 2c), with the minimum observed being ~ 40%. The reduction in mtDNA correlated with the antisense vector copy number (R^2^ = 0.9298), while the amount of mtDNA in vector controls was unchanged. The reduction in mtDNA copy number as a result of *twm1* antisense inhibition clearly indicates that Twm1 performs an important role in maintaining mtDNA copy number in *D. discoideum*, either in the replication process or DNA maintenance. Furthermore, reduced mtDNA copy number in vivo would likely result in lowered energy production and the subsequent reduction to growth rate which directly correlates to the degree of antisense inhibition observed (Fig. 2a).

### Ethidium bromide exposure increases *twm1* expression and results in mtDNA loss from which antisense transformants are less able to recover

**Fig. 3:**
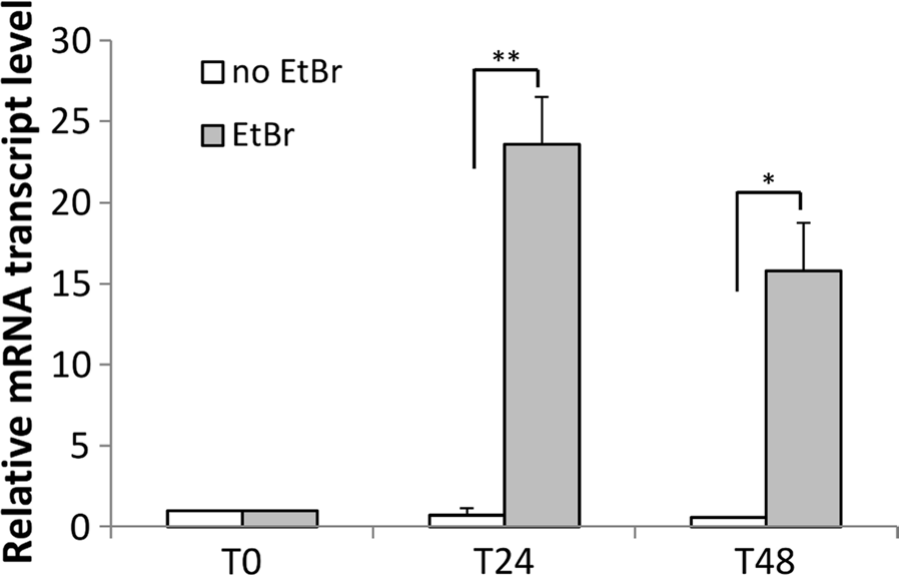
Relative *twm1* mRNA levels of *D. discoideum* AX2 in response to EtBr treatment. Initial AX2 cultures (T0) were first treated with 10 μg/ml EtBr for 24 h (T24), at which point the EtBr-containing medium was removed and replaced. Following this, cells were allowed a further 24 h to recover without EtBr (T48). A duplicate culture without EtBr treatment was used as a control. *twm1* mRNA levels were quantified with qRT-PCR and normalized against a structural gene (*tubB*). Values are relative to initial (T0) mRNA level. Error bars represent the standard error. p values were calculated using Student’s t-test (*< 0.05; **< 0.0001)

The use of ethidium bromide (EtBr) as an inhibitor of mtDNA replication has been well documented [32, 33] and is known to selectively reduce mtDNA copy number in *D. discoideum* [34]. We therefore examined the impact of EtBr exposure on Twm1 expression and function. Cultures in all experiments were treated with 10 μg/ml EtBr as this concentration does not drastically reduce the growth rate of *D. discoideum* [34] but was expected to still affect mtDNA copy number. *D. discoideum* cultures, both AX2 and *twm1* antisense transformants, were grown in the dark with EtBr for 24 h, at which point the cells were harvested, resuspended in fresh medium and allowed to recover for 24 h. AX2 mRNA levels were first examined to identify the effect of EtBr on *twm1* expression. Following 24 h treatment with EtBr, *twm1* mRNA levels had increased over 20-fold, compared to pre-treated (T0) and untreated cells (Fig. 3). After a subsequent 24 h recovery period, *twm1* mRNA levels had begun to lower, but were still 15-fold higher than that of the pre-treated cells. The increased expression of *twm1* following EtBr treatment was expected, as cells attempting to overcome inhibition of mtDNA replication would likely upregulate their replicative machinery, which presumably includes Twm1.

**Fig. 4:**
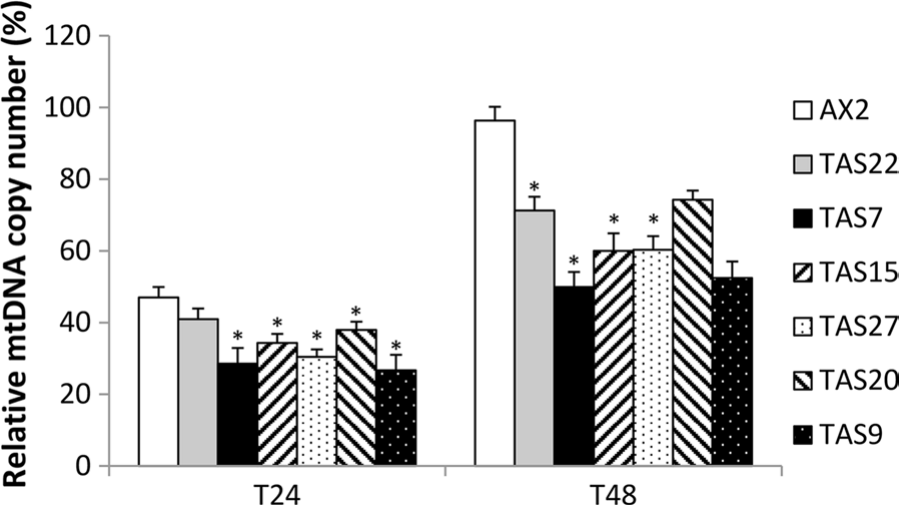
Loss and recovery of *twm1* antisense transformant mtDNA following EtBr exposure. Parental AX2 and *twm1* antisense transformants (TAS) were exposed to EtBr for 24 h, and subsequently allowed a further 24 h to recover. Relative mtDNA copy number was determined using qPCR and the single copy *tubB* nuclear gene, with each strain compared to its initial mtDNA copy number (100%). Error bars represent the standard error, while p values were calculated using the Student’s t-test (*< 0.05; **< 0.005; ***< 0.001). p values for TAS samples at T24 were determined compared to AX2 at T24. For T48 samples, the p value was calculated from the proportional increase of mtDNA from T24 to T48, given each strain’s different mtDNA copy number at T24. This data is also presented in Table 1

Parental AX2 and *twm1* antisense transformants were subsequently analyzed for the effect of EtBr on their mtDNA copy number. After 24 h EtBr treatment, AX2 mtDNA was reduced to ~ 50%, but had fully recovered following the subsequent 24 h period. However all transformants, which already possessed an initially lower mtDNA copy number than AX2, lost 60–75% of their mtDNA following EtBr exposure (Table 1; Fig. 4). Furthermore, the transformants were not able to completely restore their mtDNA levels within the 24 h recovery period, unlike AX2. This is best highlighted by antisense transformant #22, which possessed a similar initial mtDNA level (87% of AX2), due to *twm1* antisense inhibition, and lost a comparable percentage to AX2, but was only able to restore ~ 30% of its mtDNA. While the heightened percentage loss in some antisense transformants is likely due to a lower starting quantity of mtDNA, the inability to recover their mtDNA is seemingly due to the antisense inhibition of *twm1*. The recovery from this treatment was significantly reduced in most transformants, however those possessing high antisense inhibition did not have significantly impaired recovery (Fig. 4). It is likely that the elevated degree of mtDNA loss in these transformants is sufficient to allow relatively normal mtDNA replication, despite the inhibition of Twm1, while transformants which lost less mtDNA displayed defective recovery. These results suggest that while EtBr-induced depletion of transformant mtDNA is likely not affected solely by antisense inhibition (i.e. initially lower mtDNA levels), the recovery from such an event is reliant on the proper function of Twm1 in regulating mtDNA copy number.

**Table 1:** Loss and recovery of *twm1* antisense transformant mtDNA following EtBr exposure

	TAS vector copy number	Initial mtDNA (%) relative to AX2	mtDNA loss (%) after 24 h	mtDNA recovery (%) after 24 h
AX2	0	100.00	53.02 ± 2.94	49.34 ± 3.83
TAS #22	25	86.56 ± 3.98	59.03 ± 3.93	30.30 ± 2.89
TAS #7	73	78.02 ± 3.30	71.48 ± 4.37	21.40 ± 4.17
TAS #15	100	66.89 ± 4.21	65.67 ± 2.51	25.68 ± 4.86
TAS #27	112	59.56 ± 3.71	69.56 ± 2.09	29.86 ± 3.78
TAS #20	115	68.31 ± 4.84	62.04 ± 2.26	36.31 ± 2.53
TAS #9	163	39.97 ± 3.81	73.30 ± 4.29	25.75 ± 4.58

Parental AX2 and *twm1* antisense transformants (TAS) were exposed to EtBr for 24 h, and subsequently allowed a further 24 h to recover. Relative mtDNA copy number was determined using qPCR and the single copy *tubB* nuclear gene. Standard error is provided. mtDNA loss and recovery percentages are relative to each strain’s initial mtDNA copy number

### Twm1 possesses in vitro NTPase activity which is stimulated by DNA

**Fig. 5:**
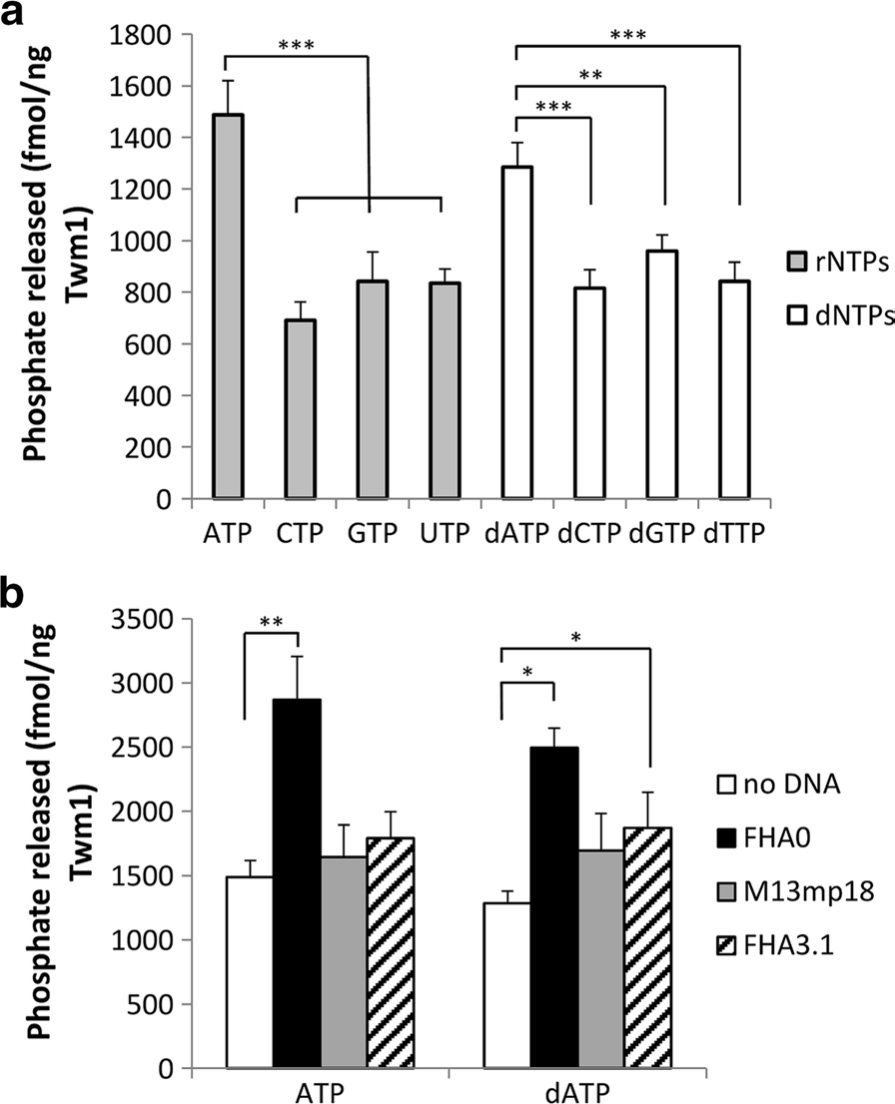
Nucleotide hydrolysis by purified Twm1 in vitro. **a** Hydrolysis of both rNTPs (grey bars) and dNTPs (white bars) by Twm1 was performed at 21 °C. **b** NTPase activity of Twm1 was also measured in the presence of linear dsDNA (FHA0; black bars), circular ssDNA (M13mp18; grey bars) or linear ssDNA (FHA3.1; hatched bars) at 21 °C. Error bars represent the standard error. p values were calculated using Student’s t-test (*< 0.05; **< 0.005; ***< 0.001). All values were normalized against no protein negative controls (empty vector purification)

The ability of helicases to unwind nucleic acids is powered by the hydrolysis of nucleotides. Therefore the in vitro NTPase activity of purified *D. discoideum* Twm1 (Additional file 2: Figure S2) was examined as an initial indicator of the recombinant protein’s activity and possible function. Twm1 hydrolyzed all nucleotides tested, but was best able to hydrolyze ATP followed by dATP (Fig. 5a). There was minimal difference between the remaining nucleotides, although Twm1 was least able to break down both dCTP and CTP (compared to other dNTPs and rNTPs, respectively). We subsequently investigated whether the protein’s NTPase activity would be stimulated by the addition of DNA, as has been observed with other Twinkle homologues. Three different templates were used: linear dsDNA (FHA0), circular ssDNA (M13mp18; New England Biolabs), and linear ssDNA (FHA3.1). The inclusion of dsDNA produced an approximately twofold increase in Twm1 hydrolysis of both dATP and ATP in vitro (Fig. 5b). Conversely, the addition of ssDNA, regardless of its structure, only marginally stimulated NTPase activity. This suggests that, like human Twinkle [3], Twm1 also favors dsDNA binding, given its significant improvement in NTPase activity compared to ssDNA.

### Twm1 functions as a 5′→3′ helicase which requires open fork-like DNA substrates

**Fig. 6:**
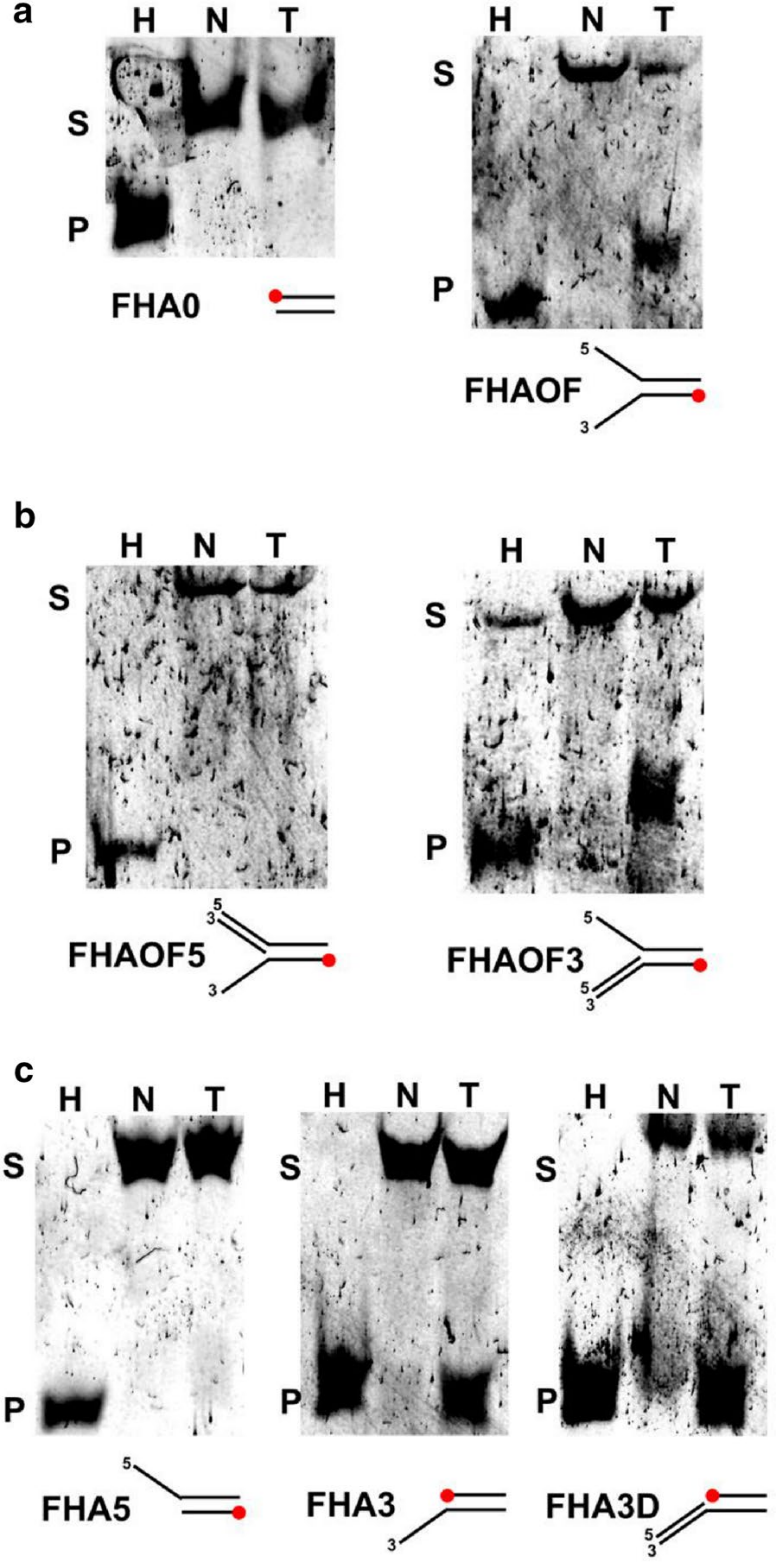
Helicase activity and substrate preference of *D. discoideum* Twm1. In vitro helicase activity of Twm1 was determined at 21 °C using various fluorescently labelled dsDNA templates (Additional file 3: Table S1B). Each DNA template was heated to 100 °C (H; first lane) and assayed using a no protein negative control (N; empty vector purification; second lane) in addition to Twm1 (T; third lane). Substrate (S) and final product (P) are indicated. Overhang polarities and FAM labels (red dots) of substrates are also indicated. **a** Helicase assay using strict dsDNA (FHA0) or open fork-like dsDNA (5′ and 3′ overhangs; FHAOF). **b** Determination of Twm1 directionality using open fork-like dsDNA with one duplex overhang (FHAOF5 or FHAOF3). **c** Overhang requirements of Twm1 were determined using dsDNA with a single ssDNA overhang (5′ or 3′; FHA5 or FHA3, respectively). Directionality of Twm1 was reconfirmed by using a duplex 3′ overhang (FHA3D)

Although Twinkle homologues function as 5′→3′ DNA helicases, they cannot unwind dsDNA substrates without particular structures; specifically open fork-like dsDNA with free 5′ and 3′ overhangs [35]. Given this, Twm1 was analyzed both for its functionality as a helicase, and for its ability to unwind particular dsDNA templates. Unlabeled and fluorescently labelled oligonucleotides were annealed together to create the dsDNA templates (Additional file 3: Table S1D), which were subsequently incubated with and without Twm1. The DNA was separated following incubation using non-denaturing gels to observe whether Twm1 was capable of unwinding the dsDNA templates. All dsDNA duplex regions and overhangs were 15 bp or nucleotides long, respectively. Much like human Twinkle [35], heterologously expressed Twm1 cannot unwind strict dsDNA, but can act upon DNA which forms an open fork (Fig. 6a), suggesting it requires both strands for its successful loading. The direction of Twm1’s helicase activity was subsequently confirmed by annealing an additional oligonucleotide to each overhang of the open fork substrate. Of these, Twm1 could only unwind the template with a duplex 3′ overhang (FHAOF3; Fig. 6b). This indicates that, like other Twinkle proteins, Twm1 functions as a 5′→3′ helicase, as its loading or unwinding is inhibited by the duplex 5′ but not the 3′ overhang.

Subsequently we decided to examine the requirement of each overhang for proper Twm1 function. Similarly to human Twinkle [35], Twm1 cannot unwind a dsDNA template with only a 5′ overhang. However, Twm1 is capable of inefficiently unwinding substrates lacking a 5′ overhang (Fig. 6c), provided that they possess a 3′ overhang, regardless of it being single- or double-stranded. This ability is in contrast to human Twinkle [35], which explicitly requires both overhangs in order to properly unwind target DNA. From this work, it can be concluded that Twm1 is a functional 5′→3′ helicase, which unwinds open fork-like DNA structures with a minimum requirement of a 3′ overhang for its loading.

### *D. discoideum* Twm1 is a functional primase in vitro

**Fig. 7:**
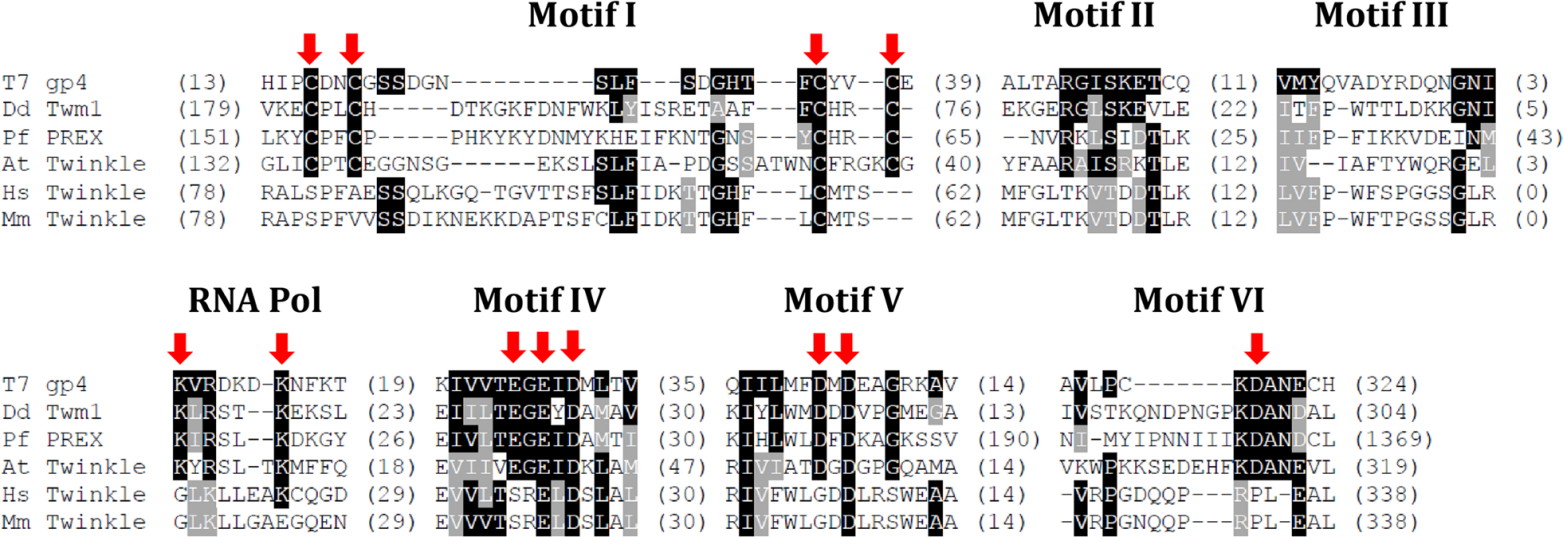
Alignment of primase motifs from bacteriophage T7 gp4 and various Twinkle helicase homologues. Conserved primase motifs (I-VI and RNA polymerase basic; RNA Pol) are those observed by Ilyina, Gorbalenya [64] and Shutt and Gray [10], while the critical residues for T7 gp4 primase activity [36, 37] are shown (arrows). Identical and similar residues (compared to the T7 gp4 sequence) are shaded in black and grey, respectively. Parentheses indicate the number of residues flanking each motif. Proteins from the following sources were used: *Arabidopsis thaliana* (At; NP_849735), *Dictyostelium discoideum* (Dd; XP_636842), *Homo sapiens* (Hs; NP_068602), *Mus musculus* (Mm; NP_722491), *Plasmodium falciparum* (Pf; XP_001348285) and bacteriophage T7 (NP_041975)

Some members of the Twinkle family of helicases are predicted to possess active primase domains, as seen in the *A. thaliana* and *P. falciparum* homologues [11, 12]; however this function is purportedly absent in human Twinkle [3] and has been hypothesized to be lost in other metazoans [10]. To better understand the function of this domain in Twinkle helicases we examined *D. discoideum* Twm1. Comparison of the T7 gp4 and Twinkle helicase sequences revealed that non-metazoan homologues have retained many of the residues crucial for gp4 primase activity, unlike those from the metazoan lineage [10]. While the putative primase domain of Twm1 is moderately homologous to T7 gp4 (Fig. 7), it retains all of the essential residues identified in gp4 [10, 36, 37]. This suggested that Twm1 was likely to have retained its primase activity, unlike metazoan Twinkle homologues.

**Fig. 8:**
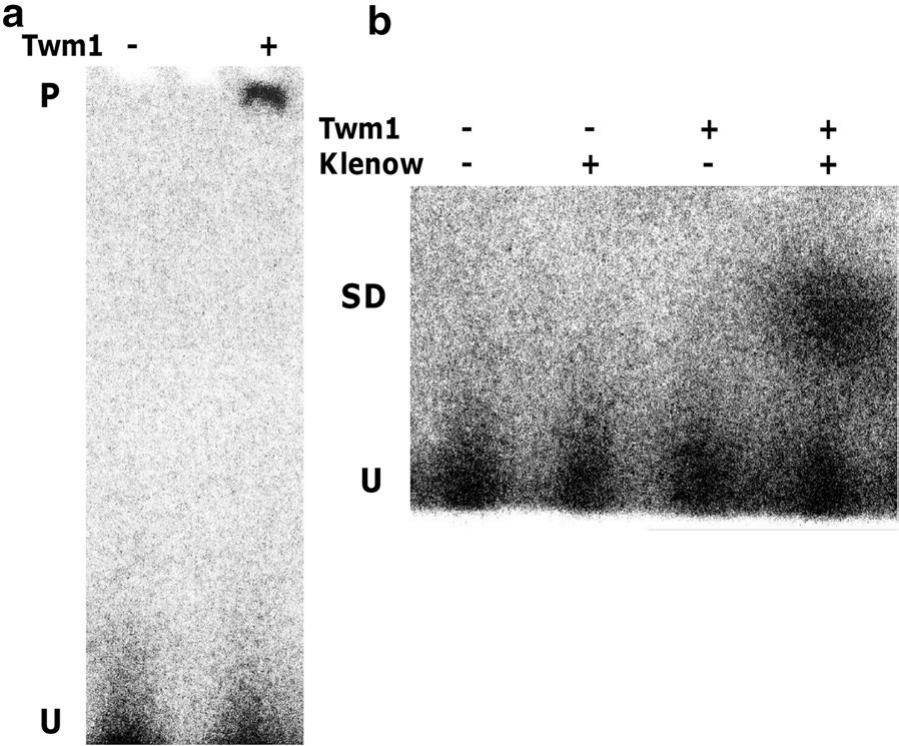
In vitro primase activity of purified *D. discoideum* Twm1. **a** Recombinant Twm1 was tested for primase activity in vitro using radiolabelled nucleotides and a circular ssDNA template (M13mp18). Following incubation, samples were run under non-denaturing conditions in order to visualise any synthesized RNA primers annealed to the template (P). Disassociated primers were too small to be differentiated from unincorporated nucleotides (U) under denaturing conditions. **b** The primase assay was repeated with the addition of Klenow DNA polymerase to determine whether any generated primer could be used for DNA synthesis. Signals were observed above the unincorporated nucleotides (U) when both enzymes (Twm1 and Klenow) were included, indicating incorporation of the radiolabelled primer into larger synthesized DNA (SD). All reactions lacking Twm1 were prepared using the empty vector purification as a negative control

To confirm whether Twm1 was capable of priming DNA in vitro, circular ssDNA (M13mp18) was employed as a template for this activity. Purified recombinant Twm1 was able to synthesize an RNA primer from radiolabeled rNTPs, which was visualized annealed to the circular ssDNA template on a non-denaturing polyacrylamide gel (Fig. 8a). Due to its presumptively small size, the disassociated primer could not be visualized under denaturing conditions, given its close proximity to the unincorporated nucleotides. Subsequently, Klenow DNA polymerase was included in the reaction to determine whether the primer could be utilized by the enzyme for de novo DNA synthesis. This resulted in the incorporation of the radiolabeled primer into DNA synthesized by the polymerase (Fig. 8b), demonstrating that Twm1 is capable of priming DNA for replication. Furthermore, the ability of Twm1 to synthesize a primer using a circular template demonstrates its ability to load itself onto circular DNA in vitro without any accessory proteins.

### Twm1 promotes DNA replication in bacterio both intrinsically and sequence specifically

*Dictyostelium discoideum* Twm1 is a presumptive replicative mtDNA helicase, given its similarity to other Twinkle homologues and the results of this study. Although the requirements to simulate mtDNA replication in vitro are quite minimal [38], performing the assay can be difficult due to potential unknown protein components and the nature of the system being examined. While Twm1 itself can be heterologously purified in soluble form, this was not possible for the suspected *D. discoideum* mtDNA polymerase, PolA (data not shown). Furthermore, a single-stranded binding protein is required for most mtDNA replication systems [38]; however no candidate has been identified for *D. discoideum*. To overcome these problems, we have adapted an in bacterio approach used previously to examine mitochondrial transcription in *D. discoideum* [22, 23].

The in bacterio system employs an *Escherichia coli* BL21 (DE3) strain possessing two vectors; one serves as the template and the other encodes the protein of interest, whose gene is under the control of an IPTG-inducible promoter. While Le et al. [22] used this system to investigate transcription by the mitochondrial RNA polymerase, we adapted the system to examine mtDNA replication by Twm1. The template vector used in this experiment was pZErO-2:NCR*rnl*, which carries a non-coding region (NCR) and an adjacent segment of the *rnl* gene from the *D. discoideum* mitochondrial genome. Although a mitochondrial origin of replication has yet to be identified in *D. discoideum*, it is likely that one is located in this NCR much like the previously identified origin of transcription [22], given that the two regulatory sequences are often located in tandem [39]. This assumption is supported by the lack of other suitable NCRs in *D. discoideum* mtDNA.

**Fig. 9:**
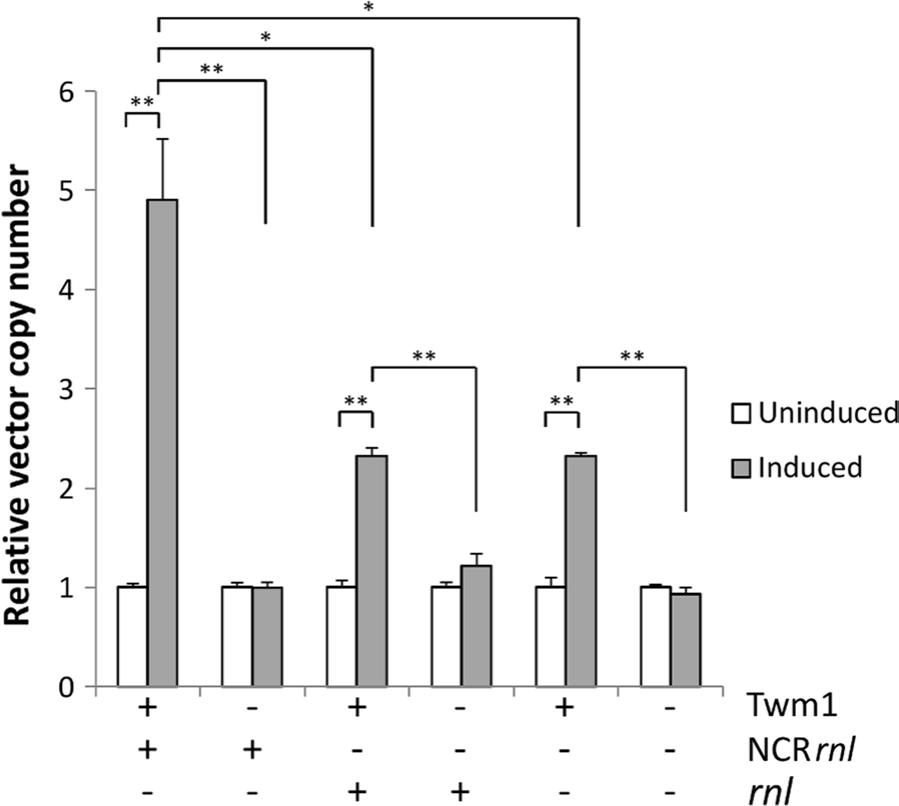
In bacterio replication of pZErO-2 by *D. discoideum* Twm1. *E. coli* BL21 strains each possessed two vectors; pET-23a:*tetA* either as an empty vector (−) or encoding Twm1 (+), and pZErO-2 with (+) or without (−) the NCR*rnl* or *rnl* region of the *D. discoideum* mitochondrial genome. Twm1 expression was induced by the addition of 1 mM IPTG. Relative pZErO-2 vector copy number (using primers for *kanR*) was quantified using qPCR against a single copy number bacterial gene, *talB*. Error bars represent the standard error. p values were calculated using Student’s t-test (*< 0.05; **< 0.001)

After the induction of Twm1 expression, we observed a fivefold increase in the copy number of the template plasmid, pZErO-2:NCR*rnl*, in bacterio (Fig. 9). A negative control strain, carrying the empty pET-23a:*tetA* expression vector, was also used to ensure that IPTG induction by itself was not able to promote endogenous replication, however plasmid copy number did not change. These results clearly demonstrate that Twm1 facilitates DNA replication in a bacterial system, presumably due to its role as a replicative mtDNA helicase in *D. discoideum*. Subsequently, we also explored whether the Twm1 activity was sequence specific by using either a template vector lacking the *D. discoideum* NCR (pZErO-2:*rnl*) or one lacking *D. discoideum* mtDNA entirely (pZErO-2). Following Twm1 induction, we observed a twofold increase in vector copy number regardless of the template, implying that Twm1 is intrinsically capable of promoting or participating in plasmid replication in bacterio. However, the fact that template vector replication was increased in the presence of the NCR suggests that this region likely contains an origin of replication which improves the ability of Twm1 to promote in bacterio replication. This result therefore suggests that Twm1 possesses some ability to recognize specific sequences, or that this sequence promotes Twm1 activity, which improves its promotion of DNA replication.

## Discussion

In this study we investigated the *D. discoideum* Twinkle homologue, Twm1, in order to better understand the function and evolution of this protein family outside of metazoans. Our results, suggest that Twm1 serves as a replicative mtDNA helicase in *D. discoideum* given its maintenance of mtDNA copy number in vivo (and plasmid copy number in bacterio), upregulation following inhibition of mtDNA replication (as a result of EtBr treatment) and unwinding of open fork-like dsDNA. However, this requires in vivo confirmation, and does not exclude the possibility that there is more than one replicative mtDNA helicase in *D. discoideum*. While metazoan Twinkle is purported to be the sole replicative mtDNA helicase [40], uncertainty over the model of mtDNA replication [41] brings into question the requirement of alternative helicases. Twinkle assumedly serves as the helicase in the replication complex for continuous DNA synthesis (i.e. synthesis of the leading strand), regardless of whether discontinuous synthesis (of the lagging strand, for example) also occurs in mitochondria. This assumption also applies to Twm1 based on the similarities identified in this work, although further investigation is still needed to clarify the exact mechanisms of mtDNA replication, both collectively and in *D. discoideum*.

Nucleotide hydrolysis is integral for helicase activity, however the nucleotide of choice can vary between unrelated or homologous proteins. T7 gp4, for example, hydrolyzes dTTP and dATP equivalently in the absence of DNA, but hydrolyzes dTTP threefold more when DNA is present [42]. Conversely, human Twinkle is less selective, but without the addition of DNA best hydrolyzes ATP and dATP [7]. This does not largely change with the addition of DNA [7], although the choice of template (dsDNA with overhangs) can decrease its selectivity for particular nucleotides [43]. Twm1, much like human Twinkle, is somewhat unselective in its use of nucleotides but prefers ATP and dATP (Fig. 5); this is unsurprising, given their close evolutionary relation. However, whether this divergence from the T7 gp4 extends to more distantly related Twinkle homologues, such as those from *A. thaliana* and *P. falciparum*, remains to be determined.

As a replicative 5′→3′ mtDNA helicase, Twinkle acts upon dsDNA with overhangs on both strands, akin to an open replication fork [35]. However, we found that while Twm1 readily unwinds these structures, it is also inefficiently capable of unwinding dsDNA with only a 3′ overhang (Fig. 6c). The apparent rate at which this occurred was low, as this was only observed after extended incubation times. This observation suggests that the substrate requirements of metazoan Twinkle are more stringent than those of other homologues, such as Twm1. One potential explanation for this disparity is that the conditions employed in previous work (e.g. incubation time) did not suit this inefficient process, leaving this activity undetected. It is also possible that this difference is due to divergences between the Twinkle homologues in different organisms. Given the importance of the Twinkle N-terminal region for proper loading and the lack of primase activity in metazoans [3, 10], the difference in substrate unwinding could also be due to sequence divergence upstream of the conserved helicase domain. Whether this activity is significant in vivo also remains to be addressed, as a possible role of this function is not as readily apparent as the unwinding of open forks. This could however support the suggestion that Twinkle is involved in recombinational repair [43], or otherwise might implicate it in alternate mtDNA maintenance processes, such as the resolution of replication fork arrest or lagging strand synthesis/maturation.

In this study we have also shown that *D. discoideum* Twm1 acts as an active primase in vitro (Fig. 8); this is the second report of primase activity in a mitochondrial Twinkle helicase [11], and the first outside plants. Based on its ability to synthesize a primer which can be used to initiate replication in vitro, it can be assumed that Twm1 performs this function during in vivo mtDNA replication. Considering this, it is of interest to note that *D. discoideum* is closely related to the opisthokont lineage, which includes metazoans, whose Twinkle proteins have lost their primase activity, and fungi, who have lost Twinkle entirely [10]. The retention of this function in Twm1 (and also in disparate organisms, such as *A. thaliana*) supports the notion that primase activity, while lost in metazoans [10], is conserved across Twinkle homologues (Fig. 7).

Human mtDNA and the T7 bacteriophage genome are both primed for replication by their respective RNA polymerase [15, 16, 44]. While in T7 the strand-coupled mechanism of DNA replication is well documented [45], there are three models of mtDNA replication which have been demonstrated in human mitochondria [41], although primer formation during replication initiation has been well attributed to the RNA polymerase [46]. In T7, gp4 is responsible for the generation of primers during lagging strand synthesis [45]. While the identity of an analogous primase in human mitochondria has yet to be determined, a candidate was recently identified; PrimPol, a primase–polymerase [47]. While this protein is an active primase [47] it is theorized to serve as a supportive enzyme, as it is non-essential and actually decreases mtDNA copy number [48, 49]. Hence, it is possible that the RNA polymerase still functions to completely prime human mtDNA; however this might not be the case in other organisms which possess a Twinkle homologue with functional primase activity, such as *D. discoideum*. It is therefore possible that primer synthesis for mtDNA replication is carried out strictly by Twm1, or in conjunction with an RNA polymerase, although this remains to be elucidated alongside clarification of the mechanism of mtDNA replication.

The role of the mitochondrial RNA polymerase in primer synthesis links the initiation of mtDNA replication to transcription [15], much like T7 DNA replication. As this has only been demonstrated in metazoans, it may well be different in other organisms, due to the differences observed between Twinkle homologues. The presence of non-metazoan Twinkle primase activity suggests that the RNA polymerase might not be required for primer formation, which could also extend to the initiation of replication; this remains to be explored, as it is not known whether the *D. discoideum* RNA polymerase is capable of priming DNA. However, should it be incapable of this function, then the mtDNA replication machinery would require the ability to initiate replication independently; this may be facilitated by the ability of Twinkle to recognize specific sequences observed in this study (Fig. 9). While this result suggests that an origin of replication is located in the NCR of the *D. discoideum* mitochondrial genome, the most interesting prospect from this work is the ability of Twm1 to specifically recognize this site and potentially drive the initiation of replication. Taken in conjunction with its primase activity [11], its ability to load itself onto circular DNA [7] and its preference for binding dsDNA [3], this activity could potentially provide non-metazoan Twinkle with the capacity to initiate mtDNA replication alone, or in tandem with other replicative proteins, as we might have seen with Twm1 (Fig. 9). While it is possible that the promotion of DNA replication in bacterio by Twm1 was entirely due to its helicase activity complementing bacterial initiation of replication, this is unlikely given that plasmid copy number did not change solely in the presence of NCR*rnl* (without Twm1). Further work is required to better understand mtDNA replication and its initiation in non-metazoan systems, and confirm which specific functions Twinkle performs in its role as a replicative mtDNA helicase.

## Conclusions

This study highlights an interesting divergence between Twinkle homologues, and demonstrates that further work is required to better understand their function in mitochondria. We have concluded that the *D. discoideum* Twinkle homologue, Twm1, is a replicative mtDNA helicase which unwinds DNA resembling open forks and promotes DNA replication. However, Twm1 is also able to unwind substrates with only a 3′ overhang, suggesting that non-metazoan Twinkle helicases might possess less stringent substrate preferences and participate in alternate mtDNA maintenance processes beyond replication. Furthermore, the ability of Twm1 to prime DNA and recognize a potential origin of replication, suggests that Twinkle homologues outside metazoans can contribute to the initiation of mtDNA replication.

## Methods

### Plasmid constructs

The *twm1* subcellular localization construct was created by cloning a portion of *twm1* (270 bp; 1–270 bp) encoding a putative mitochondrial targeting signal into the pDV-CGFP expression vector [27] to create a *twm1*-*gfp* fusion gene. The *twm1* antisense construct was created by cloning a portion of *twm1* (315 bp; 1–315 bp) in the antisense orientation downstream of the actin 6 promoter in the *D. discoideum* expression vector pDNeo2 [50]. Primers used for amplification of both gene fragments are provided in Additional file 3: Table S1A.

For heterologous protein expression, a *twm1* gene fragment (1797 bp; 463–2259 bp) was generated which encodes a truncated Twm1 (599 amino acids; 68 kDa), lacking the first 154 and last 19 amino acids from the full length protein (772 amino acids). *D. discoideum* encodes a large number of proteins possessing asparagine-rich regions [51], such as Twm1, which is predicted to include one at each terminus that do not overlap with any predicted functional domains (Additional file 1: Figure S1A). These asparagine-rich stretches are also present in proteins from *P. falciparum* and are not predicted to perform a cellular function, but do contribute to protein aggregation [52]. Hence, these regions were omitted, along with the N-terminal mitochondrial targeting signal, to minimize expressed protein aggregation. The generated gene fragment was subsequently cloned into the pET-23a:*tetA* expression vector [53].

### *Dictyostelium discoideum* strain and culture conditions

*Dictyostelium discoideum* strain AX2, a derivative of the NC4 strain, and all subsequent transformants were grown axenically in HL-5 medium at 21 °C, shaking at 180 rpm [54, 55]. Non-axenic growth of *D. discoideum* was performed on SM agar [56] with *Klebsiella aerogenes* or *Micrococcus luteus* PRF3 lawns.

### Transformation of *Dictyostelium discoideum* with vector DNA

*Dictyostelium discoideum* AX2 was transformed via the calcium phosphate method as described previously [57] using 20 μg of vector DNA. Transformants were isolated from *M. luteus* PRF3 lawns on SM plates supplemented with 20 μg/ml G-418 [58].

### Fluorescence microscopy

To determine the subcellular localization of Twm1, *D. discoideum* transformants expressing a Twm1-GFP fusion protein, encoded within pDV-CGFP [27], were analyzed via fluorescence microscopy as described previously [59, 60], using an Olympus BX 61TRF microscope and Olympus DP80 camera, following staining with 50 nM MitoTracker Red (Life Technologies) for 1 h.

### Analysis of *Dictyostelium discoideum* growth rate on bacterial lawns

*Dictyostelium discoideum* growth was analyzed by measuring plaque expansion rate on bacterial lawns [29]. Strains of interest were collected from the leading edge of grown plaques on *K. aerogenes* lawns and inoculated on normal agar plates with pregrown *E. coli* B2 lawns. Plaque diameter was measured every 8 or 16 h for 7 days to determine the mean plaque expansion rate (mm/h). Each strain was analyzed twice with three biological replicates in each experiment.

### Quantitative PCR (qPCR)

Copy numbers of the *twm1* antisense construct (in *D. discoideum* transformants), the *D. discoideum* mitochondrial genome and the pZErO-2 vector (Invitrogen) were determined using qPCR. Reactions were performed using SsoAdvanced™ Universal SYBR^®^ Green Supermix (Bio-Rad), total gDNA and appropriate gene-specific primers (cloned portion of *twm1* for antisense construct; *rns* for mtDNA; *kanR* for pZErO-2). Total gDNA was extracted using DNAzol^®^ (Astral Scientific) as per the manufacturer’s instructions. Cycling conditions were as follows: initial denaturation at 98 °C for 5 min followed by 50 cycles of denaturation at 98 °C for 10 s then annealing and primer extension at 60 °C for 30 s in an iCycler iQ^®^ (Bio-Rad). All samples were normalized to a single copy nuclear (*tubB*) or chromosomal gene (*talB*) for *D. discoideum* or *E. coli*, respectively. At least three biological replicates were analyzed in triplicate for each experiment. p values were calculated using the Student’s t-test function in Microsoft Excel. Primers used are listed in Additional file 3: Table S1B.

### Quantitative reverse transcription PCR (qRT-PCR)

The mRNA transcript levels of *twm1* in *D. discoideum* cells were determined using qRT-PCR. RNA was initially DNase treated using the TURBO DNA-*free*™ Kit (Life Technologies). Reactions were performed using iScript™ One-Step RT-PCR Kit with SYBR^®^ Green (Bio-Rad), total RNA extracted from *D. discoideum* AX2 or antisense transformants, and primers specific to *twm1*. Cycling conditions were as follows: cDNA synthesis at 50 °C for 10 min, reverse transcriptase inactivation at 95 °C for 5 min followed by 60 cycles of denaturation at 95 °C for 10 s then annealing and primer extension at 60 °C for 30 s in an iCycler iQ^®^ (Bio-Rad). Negative controls without the addition of reverse transcriptase were performed to discount gDNA contamination following DNase treatment. All samples were normalized to a nuclear structural gene (*tubB*). At least three biological replicates were analyzed in triplicate for each experiment. p values were calculated using the Student’s t-test function in Microsoft Excel. Primers used are listed in Additional file 3: Table S1B.

### Exposure of *Dictyostelium* cells to ethidium bromide

In order to induce and assay mtDNA damage, AX2 and transformant *D. discoideum* cultures were diluted to 10^5^ cells/ml and grown in the dark for 24 h with 10 μg/ml EtBr. After treatment the cells were pelleted, resuspended in fresh HL-5 and allowed to recover for a further 24 h. Aliquots of cells were harvested by centrifugation before and after treatment and following recovery. Total DNA and RNA were extracted using DNAzol^®^ (Astral Scientific) and TRIzol^®^ (Life Technologies), respectively, as per the manufacturers’ instructions. At least three biological replicates were analyzed in triplicate for each experiment.

### Expression and purification of recombinant Twm1

A pET-23a:*tetA* expression vector encoding Twm1 was transformed into *E. coli* BL21 (DE3) cells (Life Technologies) for heterologous expression and purification. A strain containing the empty expression vector was used concurrently (and for all subsequent in vitro assays) as a control for the purified recombinant protein. Cells were grown at 37 °C in LB until the OD_600nm_ reached 0.4, at which point IPTG was added to a final concentration of 1 mM to induce expression of the recombinant 6× His-tagged protein. Cells were grown for a further 4 h at 21 °C and harvested by centrifugation. Cells were resuspended in NPI-20 [pH 8.0, 50 mM NaH_2_PO_4_, 300 mM NaCl, 20 mM imidazole] and incubated with 1 mg/ml each of lysozyme and DNase on ice for 1 h. Cells were disrupted by sonication for 15 min and subjected to centrifugation at 4 °C for 30–60 min. The supernatant was then added to 1 ml Protino^®^ Ni-NTA agarose beads (Macherey-Nagel) and mixed on ice for 1 h to allow protein binding. The suspension was then added to a poly-prep column (Bio-Rad), the supernatant was allowed to flow through and the agarose beads were washed 5 times with NPI-50 [pH 8.0, 50 mM NaH_2_PO_4_, 300 mM NaCl, 50 mM imidazole]. The protein was then eluted with NPI-250 [pH 8.0, 50 mM NaH_2_PO_4_, 300 mM NaCl, 250 mM imidazole]. Protein purity was analyzed by gel electrophoresis and peak fractions were combined. Eluted protein was concentrated and dialyzed using Amicon Ultra-0.5 ml 10 K centrifugal filters (Millipore). Final protein concentration was determined via the Bradford method [61].

### Preparation of dsDNA templates

All dsDNA substrates for in vitro assays were prepared by combining oligonucleotides of equal concentrations in annealing solution [20 mM Tris-HCl (pH 8.0), 50 mM NaCl]. Mixtures were heated at 95 °C for 5 min and allowed to cool at room temperature for at least 1 h. Oligonucleotide sequences and dsDNA substrates are provided in Additional file 3: Tables S1C and D, respectively.

### NTPase assay

The in vitro NTPase activity of Twm1 was determined using a protocol adapted from Jemt, Farge [7]. NTP hydrolysis was measured by the release of phosphate using the malachite green phosphate assay. Reactions (20 μl) were prepared containing 20 mM Tris-HCl (pH 7.5), 4.5 mM MgCl_2_, 1 mM DTT, 1 mM NTP and 25–100 ng of purified Twm1. Reactions were prepared in the presence or absence of DNA and incubated at 21 °C for 4 h. DNA templates used were linear 15 bp dsDNA (FHA0), circular ssDNA (M13mp18; New England Biolabs) and linear 15 nucleotide ssDNA (FHA 3.1). Following incubation, reactions were diluted 2.5-fold in milli-Q sdH_2_O and dispensed into a 96 well microtiter plate, to which 100 μl of malachite green solution was added. The OD_620nm_ was measured on a CLARIOstar (BMG Labtech), and sample values were normalized based on values obtained for controls. The final concentration of released phosphate was determined from a standard curve of known phosphate concentrations. Malachite green solution and phosphate standards were prepared as described previously [62]. Experiments were repeated for each sample at least five times, with each experiment performed in triplicate. p values were calculated using the Student’s t-test function in Microsoft Excel.

### DNA helicase assay

Helicase reactions (10 µl) contained 20 mM Tris-HCl (pH 7.5), 5 mM MgCl_2_, 4 mM DTT, 100 µg/ml BSA, 3 mM ATP, 25 nM fluorescent dsDNA substrate (Additional file 3: Table S1B) and 25–100 ng purified Twm1. Reactions were incubated at 21 °C for 1–2 h routinely (or 20 h for extended incubations) and stopped with 5 µl stop solution [90 mM EDTA (pH 8.0), 40% glycerol, 10% SDS]. Products were separated on 20% non-denaturing polyacrylamide TBE gels and fluorescence was visualized using a Typhoon FLA 7000 Imaging system (GE Healthcare).

### DNA primase assay

The in vitro DNA primase activity of Twm1 was determined using a protocol adapted from Nielsen et al. [63] and Diray-Arce et al. [11]. Reactions were prepared with 25–100 ng purified Twm1 in 20 μl mixtures containing: 50 mM Tris-HCl (pH 8.0), 50 mM KCl, 10 mM MgCl_2_, 1 mM DTT, 100 μg/ml BSA, 0.2 mM rNTP mix (CTP, GTP and UTP), 2 mM ATP, 10 μCi/ml α^32^P-ATP and 0.25 μg M13mp18 ssDNA template (New England Biolabs). Reactions were prepared in the presence and absence of 1 U Klenow (Promega) and 0.1 mM dNTP mix (dATP, dCTP, dGTP and dTTP), and incubated at 21 °C for 1–4 h. Reaction products were separated on 8% non-denaturing TBE or 6% denaturing TBE-Urea gels (Life Technologies). The gel was then exposed to a PhosphorImager screen and visualized using a Typhoon FLA 700 Imaging system (GE Healthcare).

### In bacterio DNA replication

Analysis of Twm1’s ability to promote DNA replication in bacterial cells was based on similar work from previous studies [22, 23]. *E. coli* BL21 (DE3) cells of interest (carrying a template vector and a vector encoding the protein of interest) were grown in LB at 37 °C until an OD_600nm_ of 0.3 was reached, at which point 1 mM IPTG was added to induce heterologous protein expression, and the cultures incubated for 5 h at 21 °C. Duplicate control cultures were grown concurrently which were not induced. Aliquots of cells were harvested by centrifugation prior to and following IPTG induction and total DNA was isolated using DNAzol^®^ (Astral Scientific) as per the manufacturer’s instructions. qPCR was performed to determine copy number of the pZErO-2 vector (Invitrogen). At least three biological replicates were analyzed in triplicate for each experiment. Primers used are listed in Additional file 3: Table S1B.

## Additional files

**Additional file 1: Figure S1.** Domain architecture of Twm1 and related homologues.

**Additional file 2: Figure S2.** Purification of heterologously expressed Twm1.

**Additional file 3: Table S1.** Oligonucleotides and dsDNA substrates created for use in this study.

## Abbreviations

dsDNA: double-stranded DNA
ssDNA: single-stranded DNA
mtDNA: mitochondrial DNA
NTPase: nucleoside triphosphatase
EtBr: ethidium bromide
NCR: non-coding region
