# Supplementary material for: The Dictyostelium discoideum homologue of Twinkle, Twm1, is a mitochondrial DNA helicase, an active primase and promotes mitochondrial DNA replication
Source: BMC Mol Biol. 2018 Dec 19;19:12. doi: 10.1186/s12867-018-0114-7 (PMC6299598; doi:10.1186/s12867-018-0114-7)
Supplement: Supplementary file 1 — Additional file 1: Figure S1. Domain architecture of Twm1 and related homologues. [file 12867_2018_114_MOESM1_ESM.pdf]

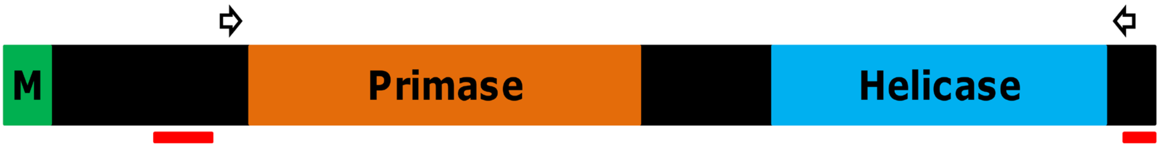


**Figure S1A: *Dictyostelium discoideum* Twm1 domain architecture.** Twm1 (772 amino acids) includes a mitochondrial targeting signal (M), a primase domain which includes a zinc finger and RNA polymerase basic motif (Fig. 7), and a helicase domain. Twm1 includes an asparagine-rich stretch at each terminus (red lines) that does not overlap with either functional domain. Arrows indicate the region of Twm1 which was heterologously expressed.

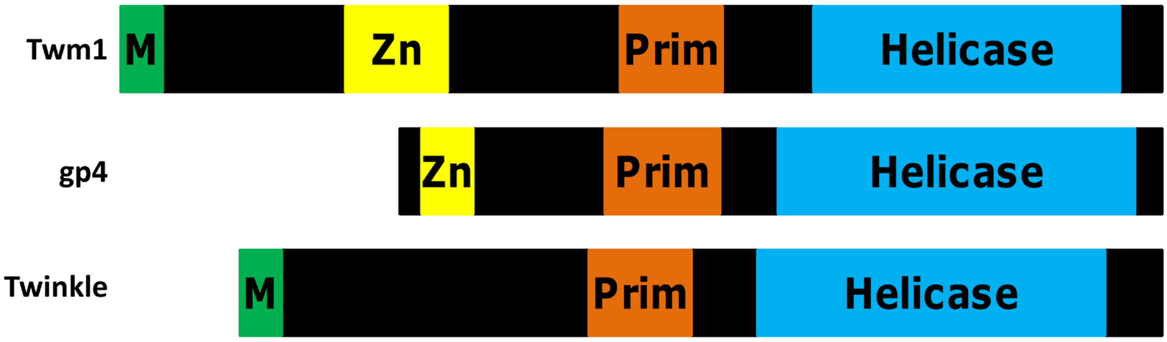


**Figure S1B: Twinkle homologues domain architecture.** *D. discoideum* Twm1 (772 amino acids), T7 gp4 (566 amino acids) and *H. sapiens* Twinkle (684 amino acids) share a conserved overall domain architecture, with some variance. All three include an active helicase domain, and a primase domain which includes a zinc finger (Zn) and other conserved motifs, including an RNA polymerase basic motif (Fig. 7). However, unlike Twm1 and gp4, Twinkle does not possess a zinc finger, and does not possess most of the residues critical for gp4 primase activity in the remaining motifs (Fig. 7). Both eukaryotic homologues include a mitochondrial targeting signal (M).
