# Supplementary material for: The Dictyostelium discoideum homologue of Twinkle, Twm1, is a mitochondrial DNA helicase, an active primase and promotes mitochondrial DNA replication
Source: BMC Mol Biol. 2018 Dec 19;19:12. doi: 10.1186/s12867-018-0114-7 (PMC6299598; doi:10.1186/s12867-018-0114-7)
Supplement: Supplementary file 2 — Additional file 2: Figure S2. Purification of heterologously expressed Twm1. [file 12867_2018_114_MOESM2_ESM.pdf]

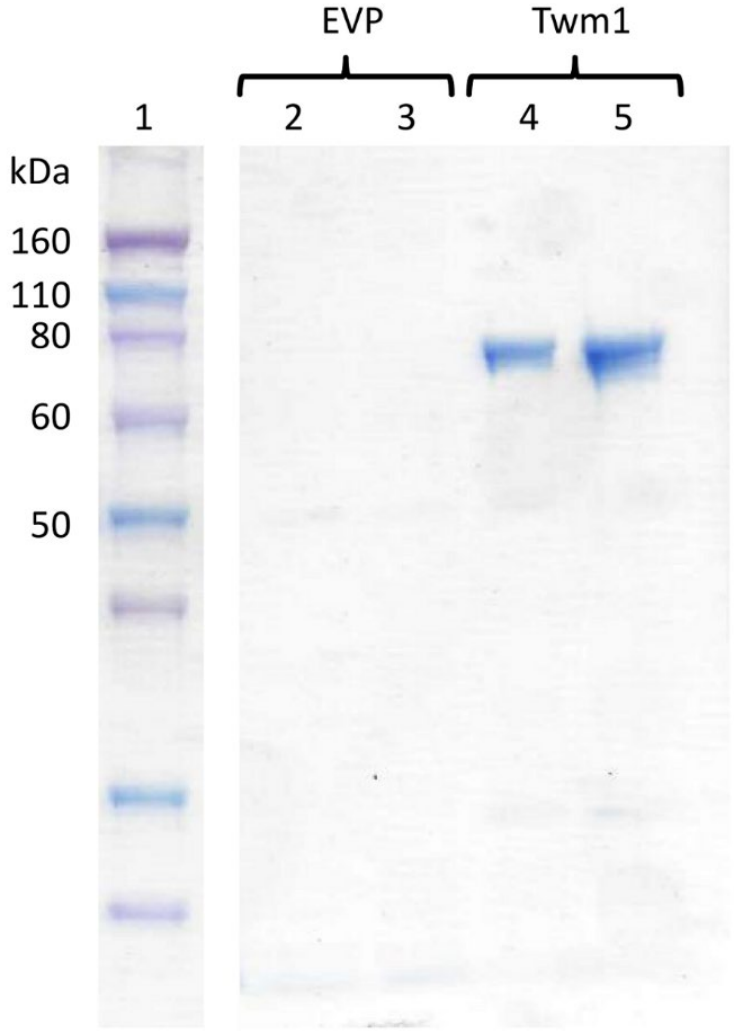

Figure S2: Purification of heterologously expressed Twm1. Coomassie blue stained SDS-PAGE of recombinant 6x His-tagged Twm1 purified from *E. coli* BL21 (DE3) cells. Sample fractions of purified Twm1 (lanes 4 and 5) were separated on 10% NuPAGE® Bis-Tris gels (Life Technologies); equivalent fractions from the empty vector purification (EVP) were also analyzed (lanes 2 and 3). Protein size was estimated using the Novex® Sharp Protein Standard (Life Technologies; lane 1).
