# Supplementary material for: The Dictyostelium discoideum homologue of Twinkle, Twm1, is a mitochondrial DNA helicase, an active primase and promotes mitochondrial DNA replication
Source: BMC Mol Biol. 2018 Dec 19;19:12. doi: 10.1186/s12867-018-0114-7 (PMC6299598; doi:10.1186/s12867-018-0114-7)
Supplement: Supplementary file 3 — Additional file 3: Table S1. Oligonucleotides and dsDNA substrates created for use in this study. [file 12867_2018_114_MOESM3_ESM.pdf]

Table S1A: Oligonucleotides used for subcellular localization and antisense construct creation.

	**Sequence (5' – 3')**	**Construct**
TMTS 5.1	GGATCCATCATGATTTCACAATGTCG	Subcellular localization
TMTS 3.1	CCACTAGTTGATGATGATGTTGTTGG	Subcellular localization
TAS 5.1	GAATTCATCGATATCATGATTTCACAATGTCG	Antisense
TAS 3.1	CTGCAGATCGATTGTTGTAGATTGTGGTGATG	Antisense

Table S1B: Oligonucleotides used for qPCR.

	**Sequence (5' – 3')**
twm1 q5.1	GTTGGTGGTGAACTTGAAAATAATC
twm1 q3.1	CTGCAGATCGATTGTTGTAGATTG
twm1 q5.2*	GGTATGGAAGGTGCTGCAAAATTCTC
twm1 q3.2*	GCATCGTTTGCATCTTTTGGACC
tubB q5.1	GCCAAAGGTGCCTCGTCATACAATC
tubB q3.1	ATTGTGTGGATCGGAAGCAGCCATC
rns q5.1	AGTTTGACTGACAGTTGGCGAAGGC
rns q3.1	TCAGACTACTCGGGTCTCTAATCCG
talB q5.1	TGGGCGAAACAGCAGAGCAACG
talB q3.1	TTGAGATACGGCCCGGAACCAG
kanR q5.1	CCACCATGATATTCGGCAAGCAG
kanR q3.1	CTCGCGCCAGCCGAACTG

* Primers used for *twm1* mRNA quantification in antisense transformants

Table S1C: Oligonucleotides used for fluorescent dsDNA substrates.

	**Sequence (5' – 3')**
FHA5.1	[6-FAM]GTAAAACGACGGCCA
FHA5.2	[6-FAM]GTAAAACGACGGCCATTTTTTTTTTTGCG
FHA3.1	TGGCCGTCGTTTTAC
FHA3.2	ACCTGTTTGGTGTTGTGGCCGTCGTTTTAC
FHA3.3	TGGCCGTCGTTTTACGTTGTGGTTTGTCCA
FHA3.4	CAACACCAAACAGGT
FHA3.5	CGCAAAAAAAAAAAA
FHA3.6	TGGACAAACCACAAC

Table S1D: dsDNA substrates created for fluorescent helicase assays.

	**Primer 1***	**Primer 2**	**Primer 3**	**dsDNA feature**	**Figure**
**FHA0**	FHA5.1	FHA3.1	N/A	None	Fig. 6A
**FHAOF**	FHA5.2	FHA3.2	N/A	Open fork	Fig. 6A
**FHAOF5**	FHA5.2	FHA3.2	FHA3.4	Open fork with duplex 5' overhang	Fig. 6B
**FHAOF3**	FHA5.2	FHA3.2	FHA3.5	Open fork with duplex 3' overhang	Fig. 6B
**FHA5**	FHA5.1	FHA3.2	N/A	5' overhang	Fig. 6C
**FHA3**	FHA5.1	FHA3.3	N/A	3' overhang	Fig. 6C
**FHA3D**	FHA5.1	FHA3.3	FHA3.6	Duplex 3' overhang	Fig. 6C

* Includes 5' 6-FAM label
